# The Centrality of Events Scale for Italian Adolescents: Integrating Traumatic Experience Into One’s Identity and Its Relation to Posttraumatic Stress Disorder Symptomatology

**DOI:** 10.5964/ejop.v14i2.1465

**Published:** 2018-06-19

**Authors:** Chiara Ionio, Eleonora Mascheroni, Paola Di Blasio

**Affiliations:** a CRIdee, Department of Psychology, Università Cattolica, Milan, Italy; Department of Psychology, Webster University Geneva, Geneva, Switzerland; The Ohio State University Wexner Medical Center, Columbus, OH, USA

**Keywords:** Centrality of Event Scale, stress, trauma, posttraumatic stress disorder, adolescents

## Abstract

Adolescents could develop areas of vulnerability, especially if they have had to deal with highly stressful and traumatic life events. Stressful experiences can work as traumatic memories that become central to one’s life and core topics for one’s identity and for the attribution of meaning to life experience. The present work evaluates (a) the internal structures of the 20-item Centrality of Event Scale in the Italian context and (b) the impact of stressful and traumatic experience during adolescence. The present work includes a convenience sample of 872 Italian adolescents -528 males, 344 females- aged between 11 and 21 years (M = 15.85; SD = 2.09). We performed a confirmatory factor analysis that confirmed a three-factor solution. Moreover, the perception of stressful event as central in the participants’ lives was significantly correlated with the presence of PTSD symptomatology, as measured by the Impact of Event Scale Revised. We found that participants with PTSD symptoms had significantly higher CES scores. These data show the validity of the CES with adolescent samples, emphasizing the sensitivity of this instrument in detecting the impact of negative life experiences even in a sample of adolescents.

Adolescence is a time of transition when several changes need to be addressed (Adams & Berzonsky, 2008; Coleman & Hendry, 1990). In addressing these changes, adolescents could develop areas of vulnerability, especially if they have had to deal with highly stressful and traumatic life events (Ionio, Olivari, & Confalonieri, 2013; Oransky, Hahn, & Stover, 2013). Ammaniti, Cimino, and Petrocchi (2007) pointed out that it is very difficult to distinguish the impact of stressful and traumatic situations and their potential role on development from the typical characteristics of the adolescent growth process. However, adolescence is recognized as a highly risky developmental period for the onset of disorders related to exposure to negative or traumatic events (Copeland, Keeler, Angold, & Costello, 2007; Ogle, Rubin, & Siegler, 2013). In fact, difficulties in psychological adaptation after a negative or traumatic event could lead to a critical development in the formation of a coherent self (Habermas & Bluck, 2000) and a definite identity (Ogle, Rubin, & Siegler, 2013).

Moreover, a recent study has shown that adolescents consider stressful events to be central turning points that could impact their ability to build a coherent self-story at both the narrative and psychological levels (Ionio et al., 2013). In fact, many previous studies have shown the important role of stressful and traumatic experiences on the construction of the self and of autobiographical memories (Berntsen & Rubin, 2006). In addition, previous research has underlined that the effects of negative events are amplified by the presence of some psychological difficulties usually experienced by adolescents (Sutherland & Bryant, 2005). Different studies have suggested that negative experiences may become central turning points in one person’s life by organizing life experiences and autobiographical memories (Berntsen, Willert, & Rubin, 2003) and becoming central to the process of building one’s personal identity and for the attribution of meaning to life experiences (Vagos, da Silva, Brazão, & Rijo, 2018).

## The Centrality of Event Scale: Theoretical Background and Prior Studies

Research on the impact of stressful or traumatic experiences on the building of an individual’s self and identity has pointed out that traumatic memories are more accessible than other kinds of memories (Reynolds & Brewin, 1998, 1999). In particular, Berntsen and Rubin (2006) underlined that memories linked to negative and potentially traumatic events are more intrusive and recurrent and more frequently recalled and relived (Berntsen, 2001). Moreover, these memories are associated with evaluative thoughts about the trauma (Reynolds & Brewin, 1998, 1999) and rumination (Nolen-Hoeksema & Morrow, 1991). Trauma-related memories are also usually better remembered than other autobiographical events (Berntsen, 2001; Porter & Birt, 2001; Reviere & Bakeman, 2001; Rubin, Feldman, & Beckham, 2004).

All of these characteristics have led Berntsen and Rubin (2006) to better investigate how stressful and traumatic events may become central to an individual’s identity over time. In their studies, Berntsen and Rubin (2006, 2007) found three aspects that could explain why an event might become central to one’s life experience. Firstly, they pointed out that memories related to stressful or traumatic events can function as reference points in the attribution of meaning to past, present, and future experiences (Vagos et al., 2018). Secondly, this theoretical framework considers memories of highly relevant events as potential central components of one’s personal identity. High stress and trauma are viewed as emblematic for the person’s self and life story and for self-understanding, making them different from other people (Berntsen & Rubin, 2006, 2007). Thirdly, Berntsen and Rubin (2006) believed that these trauma-related memories may become a turning point in one’s life story, according to a study that underlined how a traumatic or highly stressful event often causes deep changes in a person’s life (Janoff-Bulman, 1989).

Starting from this theoretical framework, Berntsen and Rubin (2006) designed the Centrality of Event Scale (CES) to measure the extent to which the memory of a highly stressful and traumatic event may become central to a person’s life, in accordance with the theoretical framework of the CES. Originally, Berntsen and Rubin (2006) underlined the unifactoriality of the CES; however, a subsequent factor analysis returned three factors (Berntsen & Rubin, 2007). These factors measure three ways in which a memory of a traumatic or stressful event may become interconnected with other personal and autobiographical memories. In particular, they assess the extent to which the memory of a traumatic or stressful event becomes (a) a reference point for everyday life, (b) a central component of personal identity, and (c) a turning point in the personal life story.

The first factor that emerged from Berntsen and Rubin’s (2007) exploratory analysis referred to the extent to which a trauma memory becomes a reference point for everyday inferences. In particular, previous research observed that people use salient personal memories to guide their thoughts and choices (Pillemer, 1998). In fact, it is well documented that memories of personally relevant events may function as anchor points for giving sense to other life experiences and for creating expectations for the future (Berntsen & Rubin, 2006). However, trauma-related memories may lead to unnecessary worries since they are usually highly accessible and perceived as involving several risks.

The second factor evaluated the extent to which trauma-related memories may become components of personal identity. According to theorists of autobiographical memory, the way people compose their life stories is closely related to the way they understand themselves (Fitzgerald, 1988). A stressful or traumatic experience may be perceived as emblematic for the person’s self. Thus, the trauma could become a stable characteristic of the self that is relevant in several life situations and consequently could affect one’s personal identity. If a traumatic event memory is seen as a central component of one’s personal identity, it could also be considered a central turning point in his or her life story.

In fact, the third factor measured the extent to which a trauma-related memory becomes a turning point in one’s personal life story. A traumatic or highly stressful event may cause deep changes in a person’s perspective (Janoff-Bulman, 1989), remaining accessible for years and coming to mind spontaneously in response to internal or external cues (Berntsen, 2001). It may be seen as a way of maintaining the personal internal consistency of the life story at the expense of the multiplicity of meaning that normally characterizes personal life narratives (Linde, 1993; Robinson, 1996).

The CES has proved to be a valid and reliable instrument with good psychometric properties (Groleau, Calhoun, Cann, & Tedeschi, 2013; Robinaugh & McNally, 2011; Schuettler & Boals, 2011). Moreover, different studies demonstrated that the CES scores were related to PTSD symptomatology and severity (Berntsen & Rubin, 2006, 2007), even controlling for depression, anxiety, dissociation, and self-absorption. These results show that the CES could be an effective tool to better understand and assess individual differences in PTSD. Other studies conducted on different samples—such as undergraduate students, combat veterans, and women abused in childhood (Berntsen & Rubin, 2006; Brown, Antonius, Kramer, Root, & Hirst, 2010; Robinaugh & McNally, 2011)—have reported positive relations between CES scores and PTSD symptomatology.

However, as far as we know, few studies have tested the CES in a sample of adolescents (Cunha, Matos, Faria, & Zagalo, 2012; Ionio, Mascheroni, & Di Blasio, 2018; Vagos et al., 2018). Although evaluating the centrality of events with adolescents whose identities and life stories are still developing could seem untimely, we believed that it is important to focus on this phase. In fact, studies have pointed out that meaning-making processes, as the integration of memories into the personal identity, are already present during adolescence (McLean, Breen, & Fournier, 2010; Vagos et al., 2018). In addition, adolescents have to deal with developmental challenges associated with the building of their future identity and roles (Kroger, 2004), which could be particularly demanding for adolescents who face peculiar life circumstances that may represent a central event to their personal identity and life story.

Moreover, traumatic memories are more accessible and inclusive than other memories. These characteristics of such memories may indicate that the trauma has increasingly become central to the subject’s identity over time. It is well documented that different aspects of stressful and traumatic memories, play a relevant role in the onset of PTSD across the life span (Brewin, Gregory, Lipton, & Burgess, 2010). However, these aspects of stressful and traumatic memories, such as the presence of recurrent and intrusive images, the sensory and emotional features of these type of memories and the fragmentation and disorganization relative to memories of the trauma, have generally been less widely investigated in adolescent samples (McKinnon, Brewer, Meiser-Stedman, & Nixon, 2017). Understanding the relative contributions of aspects of a stressful and traumatic event on adolescents’ identities could inform the profile of traumatic stress responses among youth.

## Aim

The main aim of the present study is to evaluate the impact of stressful and traumatic experience during adolescence.

Firstly, to this end, we evaluated the psychometric proprieties of the Centrality of Event Scale using a sample of Italian adolescents. We evaluated the internal structures of the 20-item version using confirmatory factor analysis (CFA) to test both the one-factor and three-factor solution proposed by Berntsen and Rubin (2006). As observed by Vagos and colleagues (2018), previous studies found inconsistent results when comparing CES scores between male and female participants. Thus, we investigated invariance across gender to ensure that we were assessing the same constructs across both groups and thus avoid inference problems (Chen, 2007).

Secondly, we evaluated whether a relationship existed between CES and PTSD symptomatology among a sample of Italian adolescents. More specifically, we investigated whether higher CES scores were associated with higher levels of PTSD symptomatology, as tested by the Impact of Event Scale Revised. As previously suggested by Cunha et al. (2012), we expected higher CES scores to be associated with higher levels of PTSD symptomatology. However, Cunha and colleagues (2012) only studied the correlation between the total CES and total IES-R scores. To better investigate the relationship between CES score and PTSD symptomatology, we also tested the correlations between the total CES, the CES subscales, and total IES-R. Furthermore, we expected to find significantly higher CES scores in those adolescents who obtained scores over the clinical cutoff in the IES-R.

## Method

### Participants

The current work included a convenience sample of adolescents recruited from different high schools in Italy. No adolescent refused to participate. The sample for this study included 872 Italian adolescents—528 males (60.1%) and 344 females (39.2%)—aged between 11 and 21 years (*M* = 15.85; *SD* = 2.09).

All of these participants were asked to think about the most negative event that they had experienced in their lives. This event was experienced at a mean age of 13.15 years (*SD* = 3.86), and an average of 3.24 years (*SD* = 3.85) had passed since this event. Table 1 shows information about the mean age when the negative event happened, the number of years since the negative event, and maternal and paternal occupation. Males and females did not differ on any of these variables.

**Table 1:** Information About Male and Female Samples

Characteristic	Male (*n* = 528)	Female (*n* = 344)	Difference	
			Test statistic	*p*
Age when the negative event happened, *M* (*SD*)	13.21 (3.99)	13.08 (3.72)	*t* = .327	.744
Time since the negative event, *M* (*SD*)	3.20 (3.92)	3.29 (3.78)	*t* = -.288	.821
Maternal occupation, %			χ^2^ = 5.71	.769
Office worker	23.1	25.5		
Artisan and worker	12.5	12.2		
Shopping activities	0.9	4.2		
Work in educational services	11.0	12.9		
Work in human and health services	8.4	6.1		
Manager	0.9	1.1		
Freelance	4.0	2.7		
Housewife	32.1	33.5		
Retired	0.9	1.5		
Unemployed	0.4	0.4		
Paternal occupation, %			χ^2^ = 9.11	.612
Office worker	16.1	16.2		
Artisan and worker	46.6	44.5		
Shopping activities	4.7	4.2		
Work in educational services	1.8	1.5		
Work in human and health services	3.6	6.4		
Manager	6.7	4.5		
Engineer	1.8	1.9		
Driver	4.9	6.8		
Policeman	2.5	2.6		
Freelance	9.4	7.9		
Retired	3.6	6.4		
Unemployed	0.2	0		

### Measures

#### Written Narrative

Demographic and event-related variables were collected from the adolescents’ written narratives about their most negative experience. They were free to tell their experience, without specific requests and without limit in terms of length of writing.

#### Centrality of Event Scale (CES)

The CES (Berntsen & Rubin, 2006) was used to measure the extent to which the memory of a stressful and traumatic event was central to the adolescent’s (a) life story, (b) personal identity, (c) attribution of meaning to other personal life events. These three factors are evaluated through 20 items rated on a 5-point Likert-type scale ranging from *totally disagree* to *totally agree*. In previous studies the CES showed an excellent internal consistency (Cronbach’s α = .94) (Berntsen & Rubin, 2006) and good convergent validity with the Beck Depression Inventory (BDI; Beck et al., 1988; .23, *p* < .01) and the Post-Traumatic Stress Disorder Checklist (PCL; Blanchard, Jones-Alexander, Buckley, & Forneris, 1996; Weathers, Litz, Herman, Huska, & Keane, 1994; .38, *p* < .01).

#### Impact of Event Scale Revised (IES-R)

The IES-R (Weiss & Marmar, 1997; Italian version by Pietrantonio, De Gennaro, Di Paolo, & Solano, 2003) was used to measure the presence of PTSD symptomatology among our sample of Italian adolescents. It is composed of 22 items rated on a 5-point Likert-type scale (from 0 = *not at all* to 4 = *extremely*). In particular, the items correspond directly to 14 of the 17 DSM-IV symptoms of PTSD: eight items regarding symptoms of avoidance (e.g., “I tried not to think about it”), seven items on intrusion symptoms (e.g., “I had dreams about it”), and seven items on hyperarousal symptoms (e.g., “I felt irritable and angry”). The sum of all items gives a total score ranging from 0 to 88. High levels of internal consistency have been previously reported (Cronbach’s alpha ranged from .87 to .94 for Intrusion, Cronbach’s alpha ranged from .84 to .87for Avoidance, Cronbach’s alpha ranged from .79 to .91 for Hyperarousal; Creamer et al., 2003; Weiss & Marmar, 1997). Test-retest reliability, collected across a 6-month interval, ranged from .89 to .94 (Weiss & Marmar, 1997). Similar internal consistency have been found in an Italian adolescent sample, specifically, α = .68 for Avoidance, α = .87 for Intrusion, α = .72 for Hyperarousal, and .87 for the total (Ionio, Camisasca, Milani, Miragoli, & Di Blasio, 2017). The Cronbach’s alpha reliability coefficients for the present study were: .71 (Avoidance), .88 (Intrusion), .72 (Hyperarousal), and .88 for the total.

### Procedure

The Italian translation and adaptation of the CES were authorized by the authors. The original version of the CES was translated by two experienced Italian researchers in psychology who are proficient in English. A native English speaker back-translated the scale into English. Researchers compared the original to the Italian translation and finalized the Italian version.

The university’s research ethics committee approved the current study. Informed consent from both parents of each participant was necessary to take part in the study. Informed consent for the protection of privacy (*Legislative Decree 30 June 2003, no. 196; Italian Privacy Code*) was a prerequisite to participate in the study.

### Data Analyses

The data were analyzed with the IBM SPSS Amos software (Version 23.0) and the IBM SPSS software (Version 23.0). IBM SPSS Amos was used for confirmatory factor analyses (CFAs) and for multigroup analyses. After the best internal structure solution was defined—comparing the one-factor solution and the three-factor solution—CFA was further applied separately to the combined male and female sample. The fit of the models resulting from these CFAs was considered on the basis of a two-index approach, namely using the comparative fit index (CFI) and the root mean square error of approximation (RMSEA). Conventional guidelines were followed, whereby the fit is considered adequate if CFI values are > .90 and RMSEA is < .08 (Bentler, 1990; Marsh, Balla, & Hau, 1996). Multigroup analyses were applied to gender. Following the approach used by Vagos et al. (2018) for the Portuguese validation study, we tested configural invariance, followed by metric invariance and scalar invariance. Configural invariance refers to the measurement model being an adequate fit for each group separately. Metric invariance adds to this the constraint that the items’ loading values also are of similar magnitude for the groups being compared. Scalar invariance, in turn, also constrains the intercepts of the items to be similar across groups. To determine measurement invariance, differences in the same fit indicators (CFI and RMSEA) were considered. As suggested by Vagos et al. (2018), metric invariance was determined based on ∆CFI ≤ −.01, combined with ∆RMSEA ≤ .015, whereas scalar invariance was determined based on ∆CFI ≤ −.01, combined with ∆RMSEA ≤ .015 (Chen, 2007).

IBM SPSS was used to calculate Cronbach’s alpha as representative of internal consistency and perform a *t*-test for paired samples to test for a possible relationship between CES and the presence of PTSD symptomatology, as measured by the IES-R.

## Results

Prior to the data analyses, the variables were examined for the presence of outliers, and the normal distribution of the items was tested (kurtosis and asymmetry ranging from −1 to +1).

### Factor Structure

We conducted a CFA on the one-factor solution model. It did not get an acceptable fit (AIC = 1,570.262; CFI = .881; RMSEA = .058). A CFA was then applied to the three-factor measurement model based on Berntsen and Rubin’s (2006) findings. The factor structure achieved an acceptable fit (AIC = 908.43; CFI = .912; RMSEA = .066). The factor loadings of items of the three factors in the model are shown in Table 2.

According to the three-factor measurement model for the 20-item version of Berntsen and Rubin’s (2006), Factor 1 measured the extent to which the event had become a reference point for expectations and the attribution of meaning to other personal life events, with factor loadings ranging from .60 to .78. Factor 2 measured the perception of the event as central to one’s personal identity with factor loadings ranging from .65 to .78. Factor 3 measured whether the event was perceived as a turning point in one’s life story with factor loadings ranging from .73 to .83.

Cronbach’s alpha reliability coefficients were computed. The α coefficients for the three factors were .85 for Factor 1, .82 for Factor 2, and .86 for Factor 3, which indicate acceptable reliability (Anastasi & Urbina, 1997; Kline, 2000).

**Table 2:** Factor Loadings of the Confirmatory Factor Analysis (CFA) of the Three-Factor Solution of the CES

Item	Factor 1	Factor 2	Factor 3
1	.60		
2	.66		
4	.69		
9	.67		
12	.78		
13	.77		
17	.73		
20	.71		
3		.72	
5		.78	
6		.76	
7		.65	
8		.71	
19		.76	
10			.80
14			.73
15			.81
16			.83
18			.82

*Note.* In the original article of Berntsen and Rubin’s (2006) Item 11 is not considered in items' structure.

### Multigroup Analyses

The fit indicators obtained separately for the male and female samples were acceptable (male: CFI = .903; RMSEA = .079, female: CFI = .910; RMSEA = .070), so further metric and scalar invariance across samples was tested. When applying the three-factor measurement model to gender—male and female—we found full metric invariance (∆RMSEA = .002; ∆CFI = −.01) but not full scalar invariance (∆RMSEA = .002; ∆CFI = .031).

### Centrality of Event Scale and PTSD Symptomatology

The correlations among the CES and IES-R scores are reported in Table 3. In particular, the overall scores and the three factors of the CES were significantly correlated with the presence of posttraumatic stress symptomatology of intrusion, avoidance, and hyperarousal.

**Table 3:** Correlation Among CES and IES-R

Scale	IES-R	IES-R INT	IES-R AVD	IES-R HYP
CES	.575***	.550***	.328***	.530***
CES F1	.501***	.470***	.300***	.457***
CES F2	.509***	.495***	.288***	.461***
CES F3	.559***	.537***	.302***	.534***

*Note.* IES-R = Impact of Event Scale Revised; IES-R INT = Impact of Event Scale Revised Intrusion symptomatology; IES-R AVO = Impact of Event Scale Revised Avoidance symptomatology; IES-R HYP = Impact of Event Scale Revised hyperarousal symptomatology; CES = Centrality of Event Scale; F1 = Factor 1; F2 = Factor 2; F3 = Factor 3.

***p < .001.

The participants obtained a mean score of 22.84 in the IES-R, ranging from a minimum of 0 to a maximum of 64. As suggested by Weiss and Marmar (1997), scores that exceed 24 can be quite meaningful. In particular, 42.5% of the adolescents in our sample obtained a score over this cutoff. Moreover, adolescents who obtained scores over 24 on the IES-R had significantly higher scores in each scale of the CES (see Table 4).

**Table 4:** Difference in CES Scores Between Adolescents That Obtained Scores Below and Above the IES-R Cut-Off of 24

Scale	Scores below 24 at IES-R		Scores above 24 at IES-R		*t*
	*M*	*SD*	*M*	*SD*	
CES	43.09	15.47	60.13	15.91	-9.564***
CES F1	14.03	6.35	20.34	6.92	-8.419***
CES F2	16.70	6.45	22.40	6.14	-7.917***
CES F3	12.37	4.62	17.40	5.10	-9.155***

*Note.* IES-R = Impact of Event Scale Revised; CES = Centrality of Event Scale; F1 = Factor 1; F2 = Factor 2; F3 = Factor 3.

***p < .001.

## Discussion

Different studies observed that the CES is a valid and reliable tools in order to assess the extent to which the memory of a highly stressful and traumatic event may become central to a person’s life in different types of clinical and nonclinical samples (Berntsen & Rubin, 2006; Brown et al., 2010; Cunha et al., 2012; Groleau et al., 2013; Robinaugh & McNally, 2011; Schuettler & Boals, 2011; Vagos et al., 2018). However, to our knowledge, still few studies explored the psychometric properties of this tool among adolescents, especially considering the Italian context (Cunha et al., 2012; Ionio et al., 2018; Vagos et al., 2018). Thus, the first aim of the present study was to evaluate factor structure of the CES in a sample of Italian adolescents. We chose to focus on this specific life phase since meaning-making processes start during adolescence, which are the integration of memories into one’s personal identity (McLean, Breen, & Fournier, 2010; Vagos et al., 2018) and the building of one’s personal future identity (Kroger, 2004). We tested both the one-factor and three-factor solutions, in accordance with the theoretical framework of the CES, underlying the processes that characterize the centrality of event processing in autobiographical memory organization. After verifying the normal distribution of the items, we performed CFA, which showed that the one-factor solution, although previously advocated with adults (Berntsen & Rubin, 2006), did not achieve acceptable fit for this sample.

Our analyses demonstrated that the three-factor solution based on Berntsen and Rubin’s (2006) findings was a better solution for our data. However, this solution did not reach full scalar invariance across gender. As suggested by Vagos and colleagues (2018), boys and girls may process and integrate stressful and traumatic experiences in different ways during adolescence. For example, males may generally split the negative effect of a stressful experience into each of its components, while females may have more difficulties in marking the different effects that traumatic events may have had on their life stories.

Moreover, we wanted to evaluate the impact of stressful and traumatic experiences during adolescence by exploring if a relationship existed between CES scores and PTSD symptomatology among a sample of Italian adolescents. More specifically, we investigated whether higher scores on the CES were associated with higher levels of PTSD symptomatology, as tested by the Impact of Event Scale Revised, to better understand if the centrality of stressful or traumatic events among adolescents is related to the development of PTSD symptomatology. We found a significant positive correlation between CES and IES-R scores, considering both the total scores and the subscales’ scores. According to Berntsen and Rubin’s findings (2006, 2007), our results pointed out that the mechanisms measured by the CES are important to better understand the presence of individual differences in PTSD symptomatology. Our sample comprised a population of adolescents who experienced or did not experience a traumatic event, with or without PTSD symptomatology. We found that participants with an IES-R score over the critical cutoff indicating PTSD had significantly higher CES scores compared to the others. These results indicate and increase our knowledge about the large individual differences in the development of PTSD symptomatology in response to stressful events (Brewin, Andrews, & Valentine, 2000; Ionio & Di Blasio, 2014; Ionio & Mascheroni, 2014; McNally, Bryant, & Ehlers, 2003). In particular, we confirm in our Italian sample of adolescents what was found by the authors of the CES (Berntsen & Rubin, 2006, 2007): the impact of a one-time event changes according to the individual’s perception and evaluation of the event.

This study is not without limitations, namely the fact that it did not include a wide range of ages. An important next step will be to test the invariance among different ages. Moreover, we tested a nonclinical population of adolescents, which reduces the generalizability of our findings. We believe that to better understand the centrality of events in the organization of one’s autobiographical memory and cognitive processing during adolescence, it will be necessary to validate the CES also in in clinical samples of Italian adolescents. A further next step will be to explore whether different traumatic events (for example, being sexually assaulted, child neglect and maltreatment, the death of a parent, etc.) are central to adolescents’ life story, to their personal identity, and to the attribution of meaning to other personal life events in the same or in different ways. In addition, in order to better understand the underlying psychological operations measured by the CES, an important issue for future research will be the relationship between the CES and other psychological scales that measure, for example, depression, anxiety, and maladaptive cognitive processing styles in response to stressful events.

In conclusion, our findings suggested that the CES could be a valid measure in the Italian context that is suitable for adolescents. We believe that this tool could be useful for the early identification of psychological disorders in adolescents.

## References

[r1] Adams, G. R., & Berzonsky, M. (Eds.). (2008). *Blackwell handbook of adolescence* (Vol. 8). 10.1002/9780470756607

[r2] Ammaniti M. Cimino S. Petrocchi M. (2007). Indagine sull’impatto di situazioni traumatiche in adolescenza: Screening dei problemi emotivo comportamentali, dei vissuti dissociativi e delle strategie difensive. Maltrattamento e abuso all’infanzia*,* 9(2), 11-32. doi:10.1400/93104

[r3] Anastasi, A., & Urbina, S. (1997). *Psychological testing* (7th ed.). Upper Saddle River, NJ, USA: Prentice Hall.

[r4] Beck A. T. Steer R. A. Carbin M. G. (1988). Psychometric properties of the Beck Depression Inventory: Twenty-five years of evaluation. Clinical Psychology Review, 8(1), 77–100. 10.1016/0272-7358(88)90050-5

[r5] Bentler P. M. (1990). Comparative fit indexes in structural models. Psychological Bulletin, 107(2), 238–246. 10.1037/0033-2909.107.2.238 2320703

[r6] Berntsen D. (2001). Involuntary memories of emotional events: Do memories of traumas and extremely happy events differ? Applied Cognitive Psychology, 15(7), S135–S158. 10.1002/acp.838

[r7] Berntsen D. Rubin D. C. (2006). The centrality of event scale: A measure of integrating a trauma into one’s identity and its relation to post-traumatic stress disorder symptoms. Behaviour Research and Therapy, 44(2), 219–231. 10.1016/j.brat.2005.01.009 16389062 PMC3974102

[r8] Berntsen D. Rubin D. C. (2007). When a trauma becomes a key to identity: Enhanced integration of trauma memories predicts posttraumatic stress disorder symptoms. Applied Cognitive Psychology, 21(4), 417–431. 10.1002/acp.1290

[r9] Berntsen D. Willert M. Rubin D. C. (2003). Splintered memories or vivid landmarks? Qualities and organization of traumatic memories with and without PTSD. Applied Cognitive Psychology, 17(6), 675–693. 10.1002/acp.894

[r10] Blanchard E. B. Jones-Alexander J. Buckley T. C. Forneris C. A. (1996). Psychometric properties of the PTSD Checklist (PCL). Behaviour Research and Therapy, 34(8), 669–673. 10.1016/0005-7967(96)00033-2 8870294

[r11] Brewin C. R. Andrews B. Valentine J. D. (2000). Meta-analysis of risk factors for posttraumatic stress disorder in trauma-exposed adults. Journal of Consulting and Clinical Psychology, 68(5), 748–766. 10.1037/0022-006X.68.5.748 11068961

[r12] Brewin C. R. Gregory J. D. Lipton M. Burgess N. (2010). Intrusive images in psychological disorders: Characteristics, neural mechanisms, and treatment implications. Psychological Review, 117(1), 210–232. 10.1037/a0018113 20063969 PMC2834572

[r13] Brown A. D. Antonius D. Kramer M. Root J. C. Hirst W. (2010). Trauma centrality and PTSD in veterans returning from Iraq and Afghanistan. Journal of Traumatic Stress, 23(4), 496–499. 10.1002/jts.20547 20690194

[r14] Chen F. F. (2007). Sensitivity of goodness of fit indexes to lack of measurement invariance. Structural Equation Modeling, 14(3), 464–504. 10.1080/10705510701301834

[r15] Coleman, J. C., & Hendry, L. (1990). *The nature of adolescence* (2nd ed.). Florence, KY, USA: Taylor & Francis.

[r16] Copeland W. E. Keeler G. Angold A. Costello E. J. (2007). Traumatic events and posttraumatic stress in childhood. Archives of General Psychiatry, 64(5), 577–584. 10.1001/archpsyc.64.5.577 17485609

[r17] Cunha M. Matos M. Faria D. Zagalo S. (2012). Shame memories and psychopathology in adolescence: The mediator effect of shame. International Journal of Psychology & Psychological Therapy, 12(2), 203–218.

[r18] Creamer M. Bell R. Failla S. (2003). Psychometric properties of the Impact of Event Scale – Revised. Behaviour Research and Therapy, 41, 1489–1496. doi:. 10.1016/j.brat.2003.07.010 14705607

[r19] Fitzgerald J. M. (1988). Vivid memories and the reminiscence phenomenon: The role of a self narrative. Human Development, 31(5), 261–273. 10.1159/000275814

[r20] Garante per la Protezione dei Dati Personali. (2003). *Codice in materia di protezione dei dati personali (Decreto legislativo 30 giugno 2003, n. 196)*. GU n.174 del 29-7-2003 - Suppl. Ordinarion.123.

[r21] Groleau J. M. Calhoun L. G. Cann A. Tedeschi R. G. (2013). The role of centrality of events in posttraumatic distress and posttraumatic growth. Psychological Trauma: Theory, Research, Practice, and Policy, 5(5), 477–483. 10.1037/a0028809

[r22] Habermas T. Bluck S. (2000). Getting a life: The emergence of the life story in adolescence. Psychological Bulletin, 126(5), 748–769. 10.1037/0033-2909.126.5.748 10989622

[r23] Ionio C. Camisasca E. Milani L. Miragoli S. Di Blasio P. (2017). Facing death in adolescence: What leads to internalization and externalization problems? Journal of Child & Adolescent Trauma. Advance online publication 10.1007/s40653-017-0166-8 PMC7163872 32318162

[r24] Ionio C. Di Blasio P. (2014). Post-traumatic stress symptoms after childbirth and early mother–child interactions: An exploratory study. Journal of Reproductive and Infant Psychology, 32(2), 163–181. 10.1080/02646838.2013.841880

[r25] Ionio C. Mascheroni E. (2014). L’impatto delle esperienze di vita negative della madre sul bambino. Maltrattamento e abuso all’infanzia*,* 16(3), 87-104. 10.3280/MAL2014-003006

[r26] Ionio C. Mascheroni E. Di Blasio P. (2018). Psychometric properties of the Centrality of Event Scale in Italian adolescents. Maltrattamento e Abuso all’Infanzia*,* 20(1), 67-79. 10.3280/MAL2018-001005

[r27] Ionio C. Olivari M. G. Confalonieri E. (2013). Eventi traumatici in adolescenza: Risposte psicologiche e comportamentali. Maltrattamento e abuso all’infanzia, 15(2), 87-99.

[r28] Janoff-Bulman R. (1989). Assumptive worlds and the stress of traumatic events: Applications of the schema construct. Social Cognition, 7(2), 113–136. 10.1521/soco.1989.7.2.113

[r29] Kline, P. (2000). *Handbook of psychological testing* (2nd ed.). London, United Kingdom: Routledge.

[r30] Kroger, J. (2004). *Identity in adolescence: The balance between self and other* (3rd ed.). Hove, United Kingdom: Routledge.

[r31] Linde, C. (1993). *Life stories: The creation of coherence*. New York, NY, USA: Oxford University Press.

[r32] Marsh, H. W., Balla, J. R., & Hau, K. T. (1996). An evaluation of incremental fit indices: A clarification of mathematical and empirical properties. In G. A. Marcoulides & R. E. Schumacker (Eds.), *Advanced structural equation modeling: Issues and techniques* (pp. 315-353). Mahwah, NJ, USA: Lawrence Erlbaum Associates.

[r33] McKinnon A. Brewer N. Meiser-Stedman R. Nixon R. D. V. (2017). Trauma memory characteristics and the development of acute stress disorder and post-traumatic stress disorder in youth. Journal of Behavior Therapy and Experimental Psychiatry, 54, 112–119. 10.1016/j.jbtep.2016.07.009 27467024

[r34] McLean K. C. Breen A. Fournier M. A. (2010). Adolescent identity development: Narrative meaning-making and memory telling. Journal of Research on Adolescence, 20, 166–187. 10.1111/j.1532-7795.2009.00633.x

[r35] McNally R. J. Bryant R. A. Ehlers A. (2003). Does early psychological intervention promote recovery from posttraumatic stress? Psychological Science in the Public Interest, 4(2), 45–79. 10.1111/1529-1006.01421 26151755

[r36] Nolen-Hoeksema S. Morrow J. (1991). A prospective study of depression and posttraumatic stress symptoms after a natural disaster: The 1989 Loma Prieta Earthquake. Journal of Personality and Social Psychology, 61(1), 115–121. 10.1037/0022-3514.61.1.115 1890582

[r37] Ogle C. M. Rubin D. C. Siegler I. C. (2013). The impact of the developmental timing of trauma exposure on PTSD symptoms and psychosocial functioning among older adults. Developmental Psychology, 49(11), 2191–2200. 10.1037/a0031985 23458662 PMC3806884

[r38] Oransky M. Hahn H. Stover C. S. (2013). Caregiver and youth agreement regarding youths’ trauma histories: Implications for youths’ functioning after exposure to trauma. Journal of Youth and Adolescence, 42(10), 1528–1542. 10.1007/s10964-013-9947-z 23580028

[r39] Pietrantonio F. De Gennaro L. Di Paolo M. C. Solano L. (2003). The Impact of Event Scale: Validation of an Italian version. Journal of Psychosomatic Research, 55, 389–393. 10.1016/S0022-3999(02)00638-4 14507552

[r40] Pillemer, D. B. (1998). *Momentous events, vivid memories*. Cambridge, MA, USA: Harvard University Press.

[r41] Porter S. Birt A. R. (2001). Is traumatic memory special? A comparison of traumatic memory characteristics with memory for other emotional life experiences. Applied Cognitive Psychology, 15(7), S101–S117. 10.1002/acp.766

[r42] Reviere S. L. Bakeman R. (2001). The effects of early trauma on autobiographical memory and schematic self-representation. Applied Cognitive Psychology, 15(7), S89–S100. 10.1002/acp.836

[r43] Reynolds M. Brewin C. R. (1998). Intrusive cognitions, coping strategies and emotional responses in depression, post-traumatic stress disorder and a non-clinical population. Behaviour Research and Therapy, 36(2), 135–147. 10.1016/S0005-7967(98)00013-8 9613021

[r44] Reynolds M. Brewin C. R. (1999). Intrusive memories in depression and posttraumatic stress disorder. Behaviour Research and Therapy, 37(3), 201–215. 10.1016/S0005-7967(98)00132-6 10087639

[r45] Robinaugh D. J. McNally R. J. (2011). Trauma centrality and PTSD symptom severity in adult survivors of childhood sexual abuse. Journal of Traumatic Stress, 24(4), 483–486. 10.1002/jts.20656 21793046

[r46] Robinson, J. A. (1996). Perspective, meaning and remembering. In D. C. Rubin (Ed.), *Remembering our past: Studies in autobiographical memory* (pp. 199-217). 10.1017/CBO9780511527913

[r47] Rubin D. C. Feldman M. E. Beckham J. C. (2004). Reliving, emotions, and fragmentation in the autobiographical memories of veterans diagnosed with PTSD. Applied Cognitive Psychology, 18(1), 17–35. 10.1002/acp.950

[r48] Schuettler D. Boals A. (2011). The path to posttraumatic growth versus posttraumatic stress disorder: Contributions of event centrality and coping. Journal of Loss and Trauma, 16(2), 180–194. 10.1080/15325024.2010.519273

[r49] Sutherland K. Bryant R. A. (2005). Self‐defining memories in post‐traumatic stress disorder. British Journal of Clinical Psychology, 44(4), 591–598. 10.1348/014466505X64081 16368036

[r50] Vagos P. da Silva D. R. Brazão N. Rijo D. (2018). The Centrality of Events Scale in Portuguese adolescents: Validity evidence based on internal structure and on relations to other variables. Assessment, 25, 527–538. doi:. 10.1177/1073191116651137 27197543

[r51] Weathers, F. W., Litz, B. T., Herman, D., Huska, J., & Keane, T. (1994). *The PTSD Checklist* – *Civilian version (PCL-C).* Boston, MA, USA: National Center for PTSD.

[r52] Weiss, D. S., & Marmar, C. R. (1997). The Impact of Event Scale-Revised. In J. P. Wilson & T. M. Keane (Eds.), *Assessing psychological trauma and PTSD* (pp. 399-411). New York, NY, USA: Guilford Press.

